# Effects of weight status, sex, age, sedentary behavior, pubertal status and socioeconomic status on the physical activity of children and adolescents

**DOI:** 10.1186/s12889-025-22867-1

**Published:** 2025-05-07

**Authors:** Alma Weimann, Mandy Vogel, Tanja Poulain, Wieland Kiess

**Affiliations:** 1 Leipzig Research Center for Civilization Diseases, LIFE Child, University of Leipzig, Philipp-Rosenthal-Strasse 27, 04103 Leipzig, Germany; 2 Department of Women and Child Health, Hospital for Children and Adolescents, Center for Pediatric Research (CPL), University of Leipzig, Liebigstrasse 20a, 04103 Leipzig, Germany; 3 German Center for Child and Adolescent Health (DZKJ), partner site Leipzig/Dresden, Liebigstrasse 20a, 04103 Leipzig, Germany

**Keywords:** Physical activity, Obesity, MVPA, Child, Adolescent, Accelerometer

## Abstract

**Background:**

Physical inactivity, especially in the context of the worldwide childhood obesity epidemic, is still a growing public health concern. Moreover, both physical inactivity and overweight often become ingrained and subsequently persist into adulthood. Therefore, the main purpose of this study was to investigate how weight status, sex, age, sedentary behavior, pubertal status and socioeconomic status influence physical activity levels in children and adolescents.

**Methods:**

This study utilized accelerometer data (SenseWear Pro 2, Bodymedia) to explore correlations between the outcome variable physical activity levels and the predictor variables weight status, sex, age, sedentary behavior, pubertal status and socioeconomic status. We analyzed 847 observations from 397 children and adolescents between the ages of 5.9 and 17.9 years, employing linear mixed-effect regression models. Roughly 55% of participants were categorized as overweight, 45% normal- or underweight.

**Results:**

A mere 18% of the cohort met the World Health Organization`s activity recommendations for 60 min of moderate to vigorous physical activity (MVPA) per day on weekdays. Specifically, children classified as overweight/obese participated less frequently in MVPA compared with their normal or lower weight peers. Children 14 years of age or younger with overweight or obesity were 30 min less active per day on weekends (*p* < 0.001). Furthermore, girls were less active than boys. This effect was most pronounced on weekends for those between the ages of 14 and 17.9, when girls engaged in 21 fewer min of MVPA (*p* = 0.013). Across all participants, every year older corresponded to a weekly decrease in MVPA of approximately 47 min (*p* < 0.001). Additionally, MVPA levels declined significantly with advancing pubertal stage, with postpubertal adolescents accumulating 4.1 h less MVPA than their prepubertal peers (*p* < 0.001). Low socioeconomic status was associated with an 18-minute reduction in MVPA (*p* = 0.016) on weekends, but only in boys.

**Conclusion:**

These findings demonstrate a significant association between physical activity levels and the examined predictors, highlighting the importance of addressing multiple factors to enhance physical activity engagement. The results underscore the need for a multifaceted, targeted approach at enhancing physical activity levels and improving child health, curbing the ongoing prevalence of overweight and obesity in childhood and subsequent adulthood.

## Introduction

The high prevalence of overweight and obesity (OW/OB) in Germany is concerning, with a recent study categorizing 15.4% of children and adolescents as overweight and 5.9% as obese [1]. This elevated rate poses significant health risks, including hypertension, impaired glucose tolerance, breathing difficulties, and psychological comorbidities, which can manifest from an early age and lead to the premature onset of illnesses [2, 3]. Physically active children and adolescents tend to have better overall health, encompassing physical, mental and psychosocial well-being [4–6]. Conversely, prolonged sedentary periods have been recognized as detrimental to overall health [7]. Therefore, promoting sufficient PA therefore represents a key strategy not only in addressing OW/OB but also in strengthening child and adolescent health more broadly [8].

However, previous research on the relationship between PA and weight status has yielded inconsistent results. While Mutz and Albrecht found no significant associations [9], Basterfield et al. reported lower PA levels (approximately 20% less MVPA) among boys considered OW/OB [10]. Kettner et al. suggested a positive association for both sexes [11]. These inconsistencies highlight the complexity of the factors that influence PA levels and point to the influence of numerous interrelated factors, including sociodemographic, biological and environmental determinants.

Sex differences remain a central theme in PA research. Across studies, boys are more likely to meet the WHO’s recommended 60 min of MVPA per day compared with girls [9, 11–13]. While the influence of age on PA remains debated—some authors report no association [9], others, like Finger et al., identify a consistent decline [12]—this variation may partly be explained by differences in pubertal timing. Beyond chronological age, the stage of pubertal development may shape PA behavior in important ways. The physical, hormonal and psychological changes accompanying puberty—such as shifts in body composition, self-image and motivation—may reduce engagement in PA, particularly among girls [14, 15]. Several studies have demonstrated that MVPA levels tend to decline as children enter and progress through puberty, with postpubertal adolescents often showing the lowest levels of engagement [16]. Incorporating pubertal status alongside age, sex and weight may therefore offer a more nuanced understanding of PA trajectories in youth.

Discrepancies in PA results of various studies could furthermore arise from differing study methodologies, including variations in PA assessment tools, analytical approaches and cut-points for PA levels. For instance, McIver et al. used direct observational methods and reported that children engaged in MVPA for roughly 13% of observed intervals within the home environment [17]. Similarly, Caspersen et al., using self-report surveys, found that the decline in PA levels was particularly pronounced through ages 15–18 years [18].

The socioeconomic status (SES) of children`s families has emerged as another potential determinant of PA levels. Children from lower SES backgrounds tend to engage less frequently in PA [9, 12, 19]. This trend could be related to limited access to recreational spaces [20] as well as parents’ financial limitations and time constraints [21]. Additionally, a lack of awareness of the significance of PA has been proposed [22]. Disparities in PA engagement thus appear to reflect a combination of biological, behavioral and environmental factors, all of which must be considered when interpreting observed patterns.

Our study aimed to explore the association between objectively measured PA levels and weight status in children in a large cohort of children and adolescents. In this context, we assessed the potential effects of sex, age, sedentary behavior, pubertal status and SES. We hypothesized a significant association between MVPA and weight status, with children and adolescents categorized as OW/OB engaging less frequently in MVPA. Furthermore, we anticipated MVPA levels to be higher in boys than in girls and to decrease with both increasing age and advanced pubertal maturation. Lastly, we expected lower SES and greater sedentary time to be negatively associated with PA.

## Methods

### Study design

The data for our study were collected as part of the LIFE Child Study, conducted at the Research Center for Civilization Diseases in Leipzig, Germany. LIFE Child is a population-based cohort study that combines a cross-sectional and longitudinal design to track physiological growth and development and to monitor the emergence of lifestyle-related diseases [23, 24]. Consequently, participants are encouraged to participate in yearly follow-up visits, resulting in multiple data sets per person.

### Participants

Inclusion criteria for our analysis required available data on the key metrics: MVPA measured by actometry, BMI, age and sex. A MVPA record was considered valid if it comprised at least four weekdays or two weekend days/holidays (categorized also as weekend day). Participants with sufficient data for the weekend days/holidays, the weekdays, or both were included in the study. Consequently, some participants were included in weekend analyses only, weekday analyses only, or both. If SES data or sedentary time data were missing, participants were excluded from models requiring those particular variables. Our analysis incorporated 847 observations from 397 children and adolescents between the ages of 5.9 and 17.9 years. Participants were grouped into age ranges to account for potential differences in PA patterns related to school and developmental stages: 5.9 up to 14.0 years of age and > 14.0 up to 17.9 years of age.

### Measurements

To monitor PA and sedentary behavior, we utilized SenseWear Pro 2 accelerometers (Bodymedia, SMT medical GmbH&Co based in Würzburg, Germany). Participants were instructed to wear the device for seven consecutive days, covering both school and non-school days (5 and 2, respectively). The 7-day measuring period could start at any day of the week, thus providing flexibility to account for holidays, school trips or the family’s schedule. The actometer features four sensors that measure triaxial acceleration, skin temperature, heat flux and galvanic skin response, allowing the proprietary SenseWear software to calculate activity levels on a per-minute basis.

Body weight and height were measured with standardized methods in accordance with the ISAK (International Society for the Advancement of Kinanthropometry) protocol [25], administered by trained, certified research assistants. Specifically, body weight was measured in light underclothes to an accuracy of 50 g using a “Seca 701” (Seca GmbH and Co. KG, Hamburg, Germany). This electronic scale is calibrated daily for maximum accuracy. Height was assessed using a “Dr. Keller I” stadiometer (Längenmesstechnik GmbH Limbach, Limbach-Oberfrohna, Germany) to an accuracy of 0.1 cm [26]. The BMI was computed and transformed into age- and sex-adjusted standard deviation scores (SDS) using German reference values [27, 28]. A BMI-SDS value exceeding 1.28 was classified as OW/OB, following the guidelines of the German Obesity Society and the German Society of Pediatrics and Adolescent Medicine [28].

Pubertal status was determined by trained study personnel using the Tanner staging system, a widely accepted method for evaluating secondary sexual characteristics. Physical examinations were conducted in a standardized manner, assigning a score from 1 to 5. This score is based on genital and breast development as well as pubic hair distribution, in accordance with the original criteria described by Marshall and Tanner [29, 30]. All assessments were performed under conditions ensuring privacy and participant comfort and were carried out in alignment with ethical research guidelines. The resulting Tanner classification served as the basis for grouping participants into either prepubertal (Tanner 1), pubertal (Tanner 2–4), or postpubertal (Tanner 5).

The family’s SES was determined as a composite score by combining the equivalized disposable household income, highest educational degree and occupational status of the parents. Each attribute was rated on a scale ranging from 1 to 7, with higher scores indicating higher income, educational status, or occupational status. Therefore, the SES score ranged from 3 to 21, with 3.0 to 8.4 classified as low SES, 8.5 to 15.4 as middle SES and 15.5 to 21.0 as high SES, in alignment with the classification of a nationwide and representative study [31].

### Statistical methods

Descriptive statistics included means and standard deviations for continuous variables and counts and percentages for categorical variables. The actometer data were provided as per-minute measurements by the SenseWear software. They included indicator variables for various activity states (e.g., sleeping, sitting, or lying down) and different PA levels (e.g., mild, moderate, or vigorous). Days with fewer than 960 min (16 h) recorded were excluded. Days were classified as weekend days or weekdays, with holidays and school vacations treated as weekend days. A recursive clustering method was used to define periods of being awake, sleeping, lying down (non-sleep), or not lying down, disregarding short, transient interruptions. In instances of unusual wake or sleep patterns, data were additionally verified by a study assistant and a data manager. If available, this secondary verification was supplemented by an activity protocol completed by the participants or their parents. In addition, the clustering algorithm results were subjected to random verification checks.

Following the identification of sleep-related points in time, such as bedtime, falling asleep, waking up and getting up, additional calculations were made for night-time and day-time min in absolute terms and as percentages of the day. Activity intensity levels (mild, moderate, vigorous) and per-minute step counts were also segmented and summed up for night and day categories. Data preprocessing was conducted with the R software, version 3.0.

Linear mixed-effect regression models, as implemented in the lme4 package [32], were utilized to assess associations between the dependent variable MVPA time (measured weekly and separately for weekdays and weekend days) and the predictor variables, including weight status, age, sex, SES, sedentary behavior and pubertal status. To account for repeated measurements for each participant, a random intercept was incorporated. In models with potential relationships with sex or age, we corrected for said factors. Interaction terms were explored to assess differences in associations between sex and age group. In addition, separate models were run for weekdays, weekends and the week as a whole, to account for variations in activity patterns across the week. All models were adjusted for potential confounders to ensure robust estimates of association.

The significance level was set to α = 0.05. All computations were executed with the R software, version 3.0 [33].

## Results

### Descriptive statistics

Table 1 provides an overview of the sample characteristics, including age distribution, weight status, pubertal status and socioeconomic background. The majority of participants (66%) were aged ≤ 14 years, while 34% were older than 14. Of the 397 participants, 178 or 44.8% were categorized as NW or LW (458 (54%) visits) and 219 or 55.2% were classified as OW/OB (389 (46%) visits). For the 847 observations from 397 participants, only 18% of children met the WHO’s daily-60-minute-MVPA recommendation on weekdays (151 cases), whereas 82% (696 cases) did not. Over the weekend, compliance improved, with 43% of children (364 cases) adhering to the guidelines, though 57% (483 cases) still fell short.

During the monitoring period, the median daily amount of time spent awake was 16.2 h for girls and 16.5 h for boys. Children 14 years of age or younger exhibited less daily sedentary behavior (1.3 h for boys, 3.2 h for girls on weekdays) than those older than 14 (9.3 h for boys and 12.2 h for girls on weekdays). These values reflect a large disparity between the sexes, irrespective of age, with girls engaging in more sedentary behavior than their male counterparts.

Regarding socioeconomic status the largest proportion of participants came from middle-income backgrounds (59%), followed by high-income (23%) and low-income (17%) groups. Detailed results regarding MVPA, sedentary behavior and related factors are presented in the following paragraphs.

**Table 1:** Characteristics of the sample

Characteristic	Male, *N* = 436^*1*^	Female, *N* = 411^*1*^	Overall, *N* = 847^*1*^
Age [years]	12.87 (10.75, 14.73)	12.75 (10.54, 14.76)	12.82 (10.63, 14.75)
Age groups			
≤ 14 years	289 (66%)	268 (65%)	557 (66%)
>14 years	147 (34%)	143 (35%)	290 (34%)
BMI-SDS^*2*^	0.92 (-0.04, 2.15)	1.06 (-0.01, 2.24)	1.03 (-0.03, 2.20)
Weight groups			
Participants NW/LW	235 (54%)	223 (54%)	458 (54%)
Participants OW/OB	201 (46%)	188 (46%)	389 (46%)
Socioeconomic status (SES)*			
Low	58 (14%)	82 (21%)	140 (17%)
Middle	260 (63%)	217 (56%)	477 (59%)
High	96 (23%)	90 (23%)	186 (23%)
Missing values	22	22	44
Median daily time spent awake while wearing the device on weekdays [h]*	16.46 (15.86, 17.07)	16.24 (15.67, 16.87)	16.36 (15.76, 16.99)
Median daily time spent awake while wearing the device on weekend days [h]*	15.82 (15.04, 16.72)	15.59 (14.74, 16.23)	15.71 (14.87, 16.48)
Sedentary time per weekday [h]*	3.9 (0.6, 9.1)	8.4 (1.1, 12.3)	5.8 (0.8, 11.0)
Sedentary time per weekend day [h]*	5.1 (0.8, 10.2)	9.1 (1.4, 12.3)	7.0 (0.9, 11.3)
MVPA^*3*^ per weekday [h]*	0.76 (0.48, 1.04)	0.54 (0.33, 0.79)	0.64 (0.39, 0.92)
MVPA^*3*^ per weekend day [h]*	1.15 (0.59, 1.81)	0.83 (0.38, 1.50)	0.96 (0.48, 1.64)
Missing values (weekdays)	80	64	144
Missing values (weekend days)	53	42	95
Pubertal status*			
Prepubertal	98 (34%)	68 (20%)	166 (27%)
Pubertal	142 (49%)	159 (47%)	301 (48%)
Postpubertal	47 (16%)	112 (33%)	159 (25%)
Missing values	149	72	221

^*1*^ Median (IQR); n (%)

^*2*^ Body Mass Index, age- and sex-adjusted SDS

^*3*^ moderate-to-vigorous physical activity

^*^ statistical significance reached

### Association between PA and weight status

**Fig. 1:**
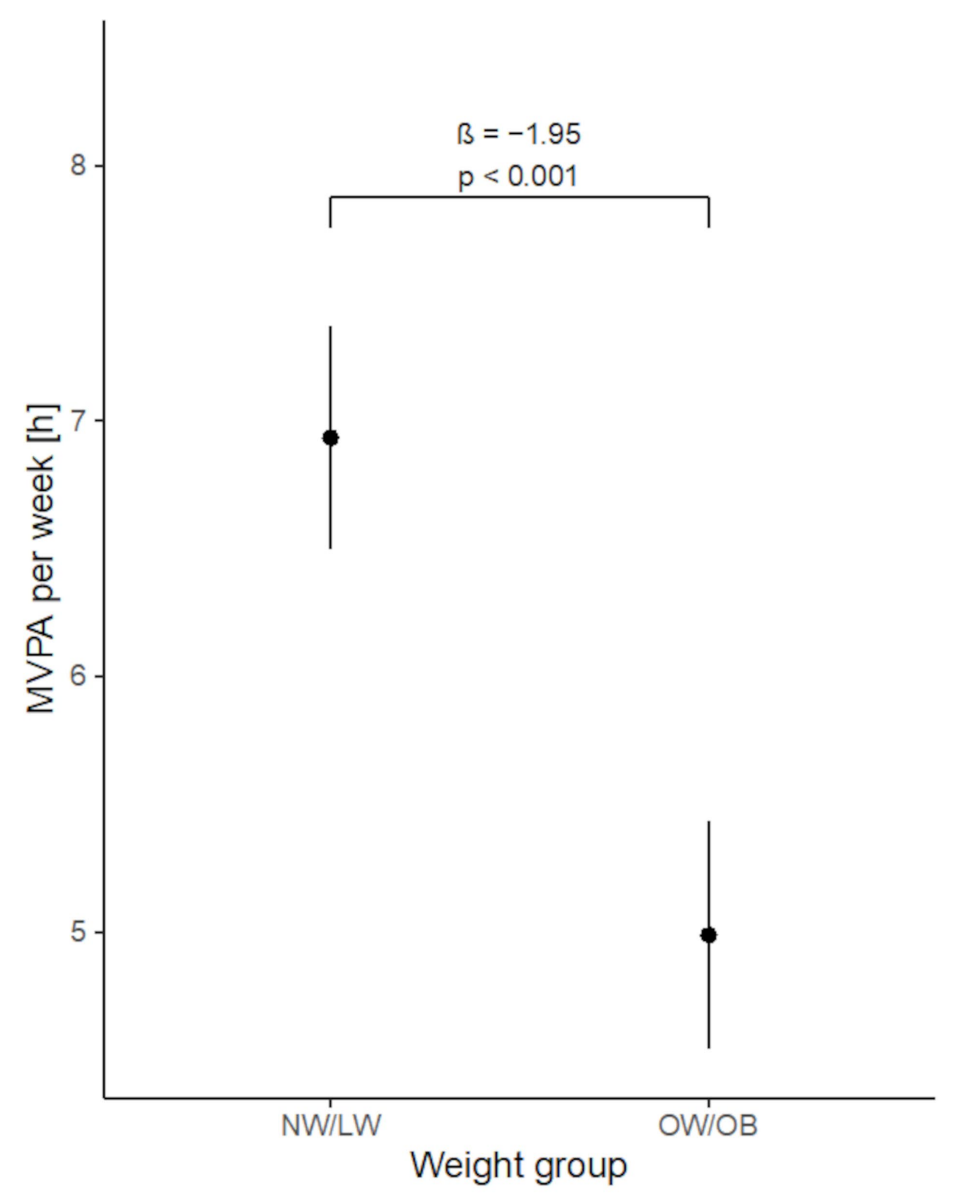
Association between MVPA and weight status (per week)

A classification of OW/OB was linked to substantially lower MVPA levels, indicating that excess weight negatively impacts physical activity engagement. Specifically, OW/OB children 14 years of age or younger participated in 1.9 h (i.e., 117 min) less MVPA per week compared with those in the NW/LW group (*p* < 0.001). This effect was similar in adolescents older than 14 up to 17.9 years of age (1.9 h or 116 min, *p* < 0.001) (Fig. 1). Notably, the reduction in PA was more pronounced during weekends for the 5.9 to 14-year-olds, with 30 min less MVPA, as detailed in Table 2.

**Table 2:** Association between MVPA and weight status in both age groups

Time period	Age group ≤ 14 years		Age group > 14 years	
	MVPA disparity OW/OB	*p*-value	MVPA disparity OW/OB	*p*-value
weekdays	− 10 min	< 0.001*	− 13 min	< 0.001*
weekend days	− 30 min	< 0.001*	− 23 min	< 0.001*
week (total)	− 117 min	< 0.001*	− 116 min	< 0.001*

* statistical significance reached

### PA’s associations with sex and age group

Girls between the ages of 5.9 and 14 participated in 104 min less MVPA per week (~ 15 min/d) compared with boys of the same age (*p* < 0.001). The gap narrowed slightly among those over 14 up to 17.9 years of age, with girls engaging in 77 min less MVPA (~ 11 min/d,

*p* = 0.060). The sex-related disparity was slightly stronger on weekends for the older group, with girls participating in MVPA for 21 fewer min per weekend day (*p* = 0.013).

**Fig. 2:**
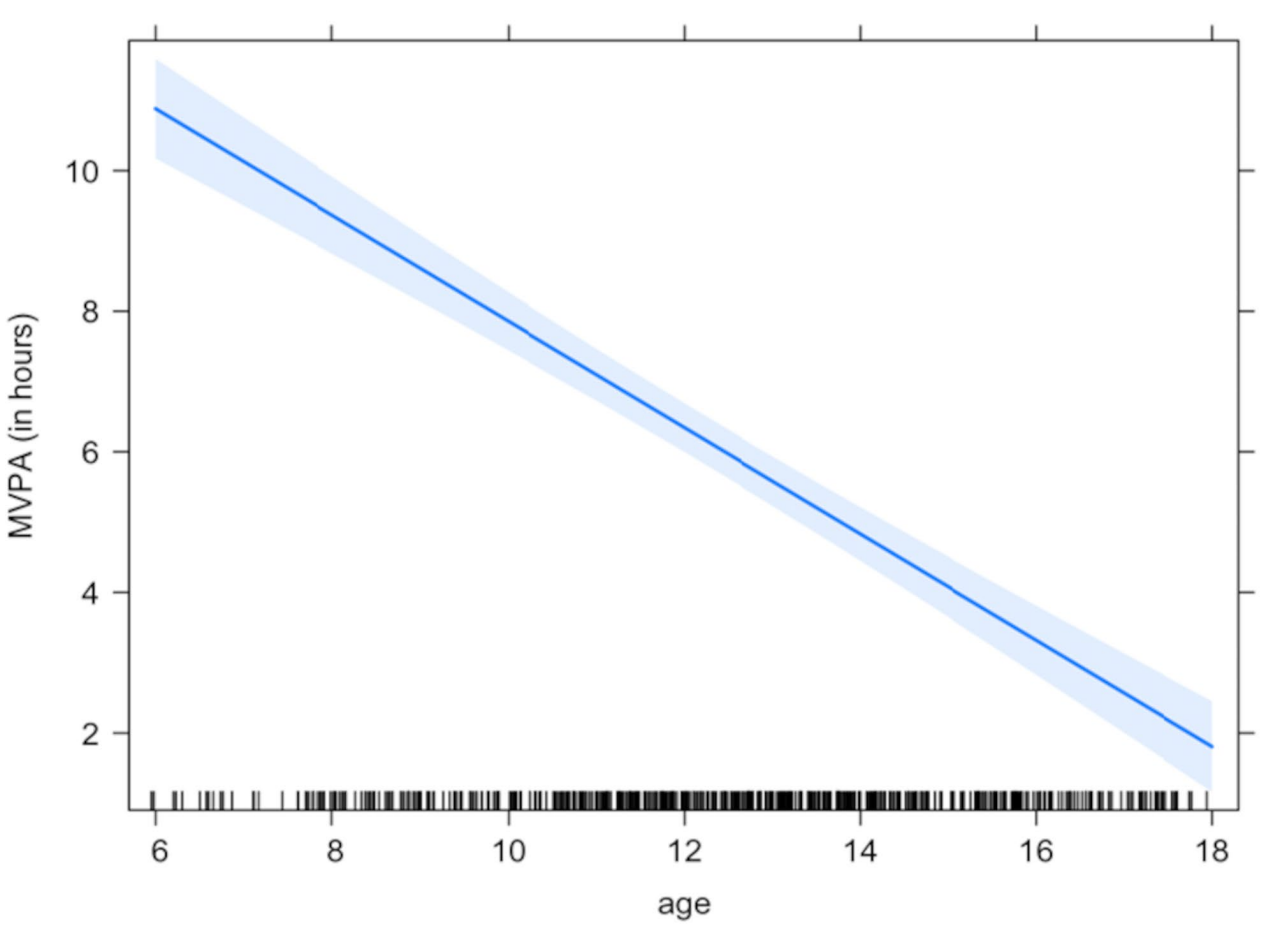
Association between MVPA and age (per week)

Furthermore, for each additional year of age, the weekly MVPA time decreased by 47 min (~ 7 min/d; *p* < 0.001) (Fig. 2). This trend was observable for both weekdays (5 min/d; *p* < 0.001) and weekends (10 min/d) but stronger on weekends (*p* < 0.001).

### Association between PA and pubertal status

**Fig. 3:**
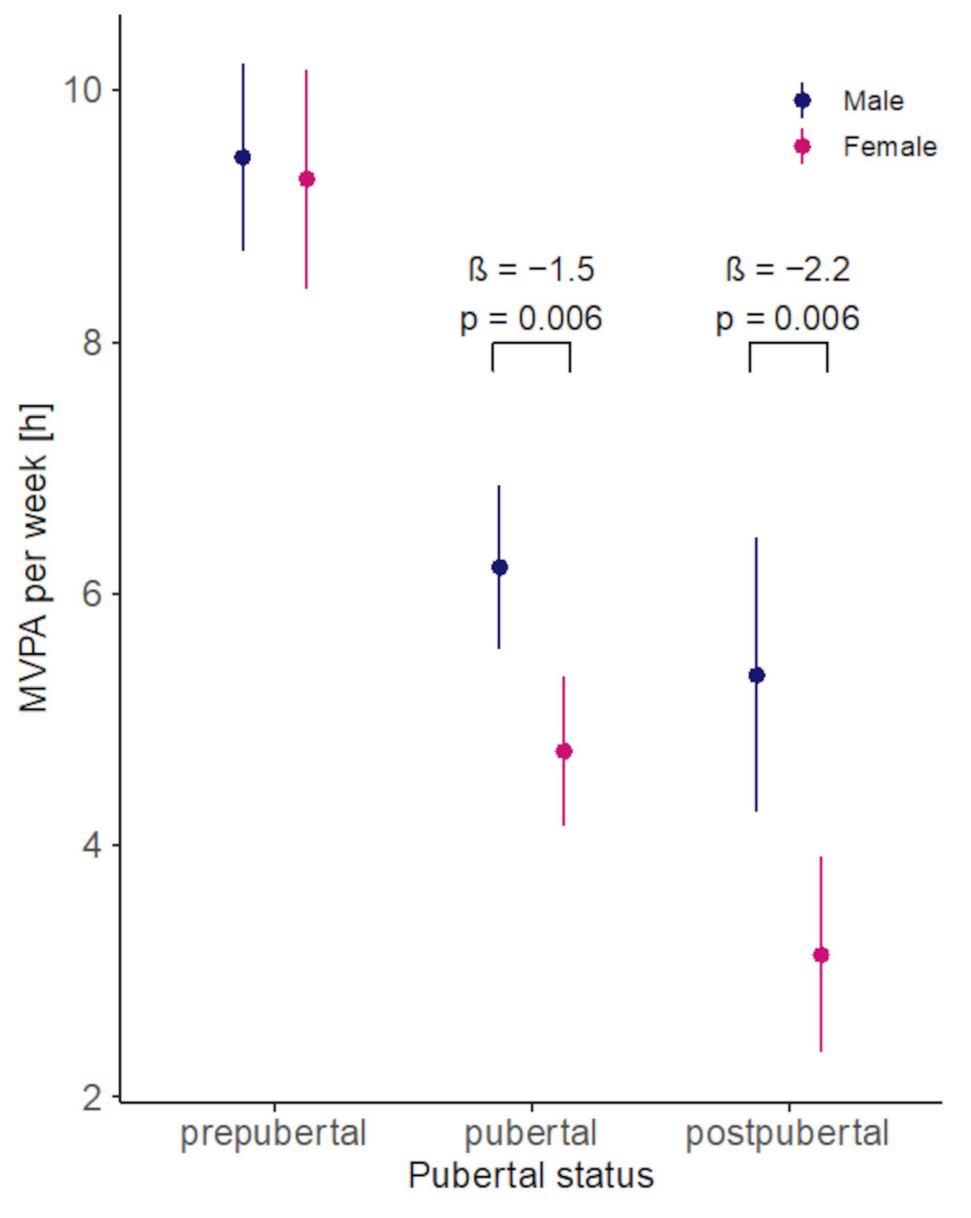
Association between MVPA and pubertal status (per week)

Our analysis based on pubertal status revealed significant differences in MVPA levels across developmental stages. Compared with prepubertal participants, pubertal children and adolescents engaged in 3.3 h less MVPA per week (*p* < 0.001), while postpubertal individuals showed an even greater reduction of 4.1 h (*p* < 0.001). Additionally, a sex-specific effect emerged: pubertal girls accumulated 1.5 h less MVPA per week than boys in the same developmental stage (*p* = 0.006). This difference persisted in the postpubertal group, with girls accumulating 2.2 h less than their male peers (*p* = 0.006) (Fig. 3).

Separate analyses for weekdays and weekends revealed that this decline was more pronounced on weekends. Postpubertal participants accumulated 45.6 min less MVPA per weekend-day compared with their prepubertal peers (*p* < 0.001), while the difference during weekdays was 28.5 min (*p* < 0.001). Notably, sex-based differences were most evident in the postpubertal group on weekends, with girls accumulating approximately 30 min less MVPA per day than boys (*p* = 0.007).

### Association between PA and SES

A correlation between MVPA and SES was present only in boys on weekends. Specifically, on weekends, boys from low SES backgrounds engaged in 18 min (*p* = 0.016) less MVPA compared with boys from middle or high SES families. Furthermore, adherence to the recommended 60 min of MVPA per day differed by SES. On weekends, approximately 60% of boys from families with a middle or high SES reached the recommended PA levels compared with only 47% of boys with a low SES background doing so (*p* = 0.09).

### Association between PA and sedentary behavior

Sedentary behavior showed a negative association with MVPA time. Each additional hour of time spent being sedentary per week corresponded to a reduction of 4 min in MVPA (*p* < 0.001). No significant differences were detected between age groups. The association was weaker on weekdays (beta = 3 min/d, *p* < 0.001) than on weekends (beta = 6 min/d, *p* < 0.001).

## Discussion

Our research examined associations between PA and weight status, age, sex, SES and sedentary behavior.

### Association between PA and weight status

Our study identified significantly lower PA levels in children with OW/OB. This observation is consistent with findings, for example, by Steele et al. [34, 35]. However, contrary results have been reported in other studies [9–11]. These discrepancies could be attributed to factors such as varying study population characteristics (e.g., a low proportion of normal weight children) or a reliance on self-reported PA times. Additionally, variations in cultural or environmental contexts across different study groups could lead to different access and attitudes towards physical activity, influencing these varying outcomes. Other factors such as school policies, local infrastructure for active commuting and varying levels of parental support may also contribute to these differences and should be considered in future research.

Less PA in children considered OW/OB may be caused by hindrances—such as impaired mobility, increased fatigue and psychosocial challenges (e.g., body image dissatisfaction and low self-esteem)—which might further discourage participation in PA [35]. Moreover, these children might have limited access to safe and appealing environments for PA and may receive relatively less encouragement from parents and peers [36]. These issues can exacerbate weight gain, perpetuating a detrimental cycle. Thus, targeted interventions should specifically address psychosocial barriers, improve environmental accessibility to PA and engage family support networks to interrupt this cycle. Additionally, it is important to explore how structured physical activity programs could be designed to cater specifically to OW/OB youth, thus providing opportunities for gradual engagement and promoting sustained participation.

Conversely, the smaller differences on weekdays may result from school hours uniformly restricting PA opportunities for children from both weight groups. However, the pronounced weekend discrepancy highlights the need for family-centered intervention strategies to promote an active lifestyle, especially for families with children considered OW/OB. Future studies could investigate effective ways to engage families, especially on weekends, such as structured family-based activities or community-based weekend programs, to enhance PA participation among these children.

### Association between PA and sex and age group

This study corroborates previous studies [9, 11–13] that have shown that girls typically engage in less PA than boys. The LOOK longitudinal study, involving 555 children between the ages of 8 and 12 years, suggested that girls’ PA levels are less influenced by socioecological factors at the individual, family, school and environmental levels. Furthermore, Telford et al. found that girls often display less favorable physiological and fitness-related characteristics (e.g., higher body fat percentages, lower cardio-respiratory fitness and lower eye-hand coordination [14]). These characteristics may not only hinder their PA engagement but could also, in turn, be influenced by lower PA levels over time, suggesting a bidirectional relationship. Addressing this bidirectional influence is crucial; interventions could simultaneously target increasing PA levels and improving physiological and motor skills through targeted physical education and structured activities specifically designed for girls. Furthermore, social norms and perceived gender roles may further impact participation rates, underscoring the importance of reshaping attitudes toward girls’ involvement in PA.

Our study found a modest sex-related divergence in PA that is more evident on weekends and among adolescents older than 14 years of age. This trend underscores the need for diverse PA incentives to engage adolescent girls, particularly during weekends. Programs tailored specifically to the interests and motivations of adolescent girls, including social and peer-supported activities, could be more effective than generalized interventions. Moreover, considering the increasing reliance on digital entertainment, creating and integrating technology-based PA solutions, such as gamified exercise programs, could enhance engagement among adolescents.

Consistent with existing research [12], our findings confirm a decline in PA with increasing age. This reduction is often attributed to puberty-related behavioral changes, such as the influence of digital media, shifting societal priorities and peer dynamics resulting in reduced unstructured playtime. Additionally, as children grow older, increased weekend schoolwork may further reduce PA. Therefore, intervention strategies aimed at adolescents must incorporate elements addressing these lifestyle factors [37], such as promoting balanced digital media use and integrating short, engaging PA breaks into weekend study routines. Future research should explore how integrating PA-friendly school policies, such as active recess initiatives, might help mitigate this decline.

### Association between PA and pubertal status

Our findings confirm a substantial reduction in MVPA levels as children transition through the stages of pubertal development. This decline is consistent with existing literature, which suggests that the onset and progression of puberty are associated with significant shifts in behavior, priorities and psychosocial dynamics that influence PA participation [15, 38]. Hormonal changes, evolving body image concerns and heightened self-consciousness may particularly deter spontaneous or structured PA, especially in girls [15]. The observed sex-specific effect, with postpubertal girls exhibiting the lowest MVPA levels, mirrors prior research highlighting adolescence as a critical risk period for reduced PA among females [39]. Social pressures, declining confidence in athletic ability and reduced access to inclusive sports opportunities may further exacerbate these disparities. Importantly, our weekday vs. weekend analysis indicates that the most pronounced declines occur during weekends, a time theoretically more open for discretionary activity. This pattern may reflect a lack of structured opportunities and motivational support outside the school setting. Interventions should therefore prioritize accessible, socially supportive and body-positive programs tailored to adolescents, particularly postpubertal girls, and emphasize weekend engagement. Incorporating peer-group-based activities, mentorship models and co-designed programming may be key strategies to sustain PA participation through adolescence.

### Association between PA and SES

In this study, we could not identify a general significant association between PA levels and the SES of a child’s family. However, we found this effect in boys, specifically on weekend days. This finding contrasts with several recent studies that generally found lower PA engagement among children from less affluent backgrounds [9, 12, 21]. Interestingly, Vorwerg et al. found a similar outcome, with a correlation in boys but not in girls [13]. It is crucial to acknowledge that, as some authors have highlighted, the inconsistent association between SES and PA is influenced by a spectrum of factors, including individual perceptions of family wealth [40, 41] and social desirability bias. Our results suggest that a lack of PA affects children across all socioeconomic groups, with an emphasis on children from low SES backgrounds. These findings further underscore the need for a more nuanced understanding of how exactly SES influences PA levels. Further research should investigate how the perception of socioeconomic status and family attitudes toward PA uniquely influence PA engagement, especially among boys, to better guide targeted interventions. Additionally, it would be valuable to assess how community resources such as access to sports clubs or recreational facilities might mediate the relationship between SES and PA participation.

### Association between PA and sedentary behavior

The observed inverse association between sedentary behavior and MVPA levels might initially appear obvious. However, it is crucial to explore this relationship beyond its apparent simplicity to understand the broader implications of sedentary behavior in our increasingly inactive lifestyles. This association is particularly relevant in Western societies, where daily routines often revolve around prolonged periods of sitting, such as in educational settings, in front of televisions, or with digital devices.

Along with other studies [16], our study shows that such lifestyle trends contribute to a decrease in PA. Despite the direct trade-off between sedentary behavior and MVPA being somewhat expected, the extent of the impact of sedentary behavior and the potential for intervention make it a significant area for research. We found that each additional hour of time spent being sedentary per weekend day corresponded to a reduction of 6 min in MVPA, indicating the substantial impact of sedentary behavior on PA levels, especially on weekends. Interventions should thus focus not only on promoting increased MVPA but also on strategies for reducing sedentary time, such as incorporating active breaks into sedentary periods or restructuring weekend routines to encourage more frequent movement. Increasing efforts to shift societal norms around prolonged sedentary time, particularly in academic settings, may further support reductions in sedentary behavior. The consistency of association between PA levels and sedentary time across age groups suggests that the detrimental effects of sedentary behavior on PA are general and consistent. Therefore, understanding these dynamics can guide the development of targeted strategies to reduce the amount of time young people spend being sedentary, thus enhancing PA levels among children and adolescents. Such interventions are vital for reversing the negative trends that have been observed and for promoting healthier, more active lifestyles.

## Strengths and limitations

The strengths of the study include the objective measurement of PA via actometry, thus avoiding the reporting bias often associated with self-report questionnaires. A major advantage of our research is the substantial and well-balanced sample size, comprising a proportionate mix of children and adolescents classified as NW/LW and OW/OB, providing robust data. The inclusion of a broad age range allowed us to assess developmental trends in PA, capturing differences across childhood and adolescence. Moreover, by analyzing PA patterns separately for weekdays and weekend-days, we were able to assess variations in activity influenced by school schedules and family environments.

However, the study has limitations, including its cross-sectional design, which limits causal inferences. Furthermore, while accelerometers provide an objective measure of PA, they have inherent limitations. The SenseWear Pro2 used in this study primarily detects motion-based activity and may therefor underestimate non-weight-bearing activities that involve minimal arm movement, such as cycling, rowing or resistance training. Moreover, the actometer cannot be worn in water, thereby excluding any water-based sports such as swimming or water ball.

Another limitation is the potential for selection bias, as can be seen in the larger number of participants with a high SES background. Although we adjusted for key confounders such as weight status, age, sex, sedentary time, pubertal status and SES, other unmeasured factors—such as dietary habits, parental influence and school-based PA policies—may also contribute to PA variations. The inclusion of these factors in future studies could provide a more comprehensive understanding of PA determinants. Finally, our study was conducted within a specific geographical region and results may not be fully generalizable to other populations from different cultural or environmental contexts.

Despite these limitations, our study provides valuable insights into the complex interplay between socioeconomic and sociodemographic factors influencing PA levels in children and adolescents, highlighting key areas for intervention.

## Conclusion

We confirmed OW/OB, female sex, older age, advancing pubertal status and low SES as risk factors for decreased PA levels using actometry in a large cohort of children.

Our research highlights a significant deficit in MVPA among children and adolescents with OW/OB, particularly evident on weekends. Furthermore, the study revealed a consistent trend of declining PA with increasing age, with modest sex differences in MVPA levels, where girls engaged in less PA than boys. Importantly, we observed a sharp decline in MVPA across advancing pubertal stages, with postpubertal participants being the least active overall. This effect was particularly pronounced among girls, indicating that pubertal development may further exacerbate existing sex-based disparities in physical activity. These findings accentuate the urgent need to design and implement practical, evidence-based interventions that are tailored to specific high-risk groups. Given that children and adolescents with OW/OB are at increased risk of both somatic and mental health issues, tailored strategies to promote PA among these groups are crucial.

The persistently low levels of PA among children and adolescents, regardless of their weight, underscores a pressing issue, as highlighted by the low rate of children meeting the WHO’s PA recommendation in our study. This calls for a shift from general recommendations to structured, accessible and engaging physical activity opportunities that fit into children’s daily routines.

This study’s results indicate that children are less active on weekdays compared with weekends. Therefore, strategies for increasing PA should focus more on weekdays, such as offering additional sports programs after classes and integrating active breaks into the school day to reduce sedentary time. Adapting sports classes to consider the children’s and adolescents’ preferences and current trends may further enhance participation and motivation. Furthermore, improved access to recreational facilities and active commuting options could play a critical role in increasing PA engagement among youth. Given the sharp decline in PA during and after puberty, interventions should particularly focus on adolescents, especially girls, during this transitional period. By creating more opportunities for young people to engage in PA both during the week and on weekends, a substantial impact could be generated for both preventing and addressing childhood obesity.

Future research should continue to explore the multifactorial influences affecting PA and evaluate the effectiveness of various intervention strategies, ensuring they are customized, adaptable and scalable to different populations and settings.

## Data Availability

The dataset analyzed in this study cannot be shared publicly due to ethical and legal restrictions. The LIFE Child Study collects potentially sensitive information. Publishing the data is not covered by the informed consent provided by the study participants. In accordance with the LIFE Data Protection Concept, all researchers (internal and external) interested in accessing data must sign a project agreement. Researchers may contact the LIFE Child Study by writing to forschungsdaten@medizin.uni-leipzig.de to request access.
